# Use of serial analysis of gene expression to reveal the specific regulation of gene expression profile in asthmatic rats treated by acupuncture

**DOI:** 10.1186/1423-0127-16-46

**Published:** 2009-05-06

**Authors:** Lei-Miao Yin, Gong-Hao Jiang, Yu Wang, Yan Wang, Yan-Yan Liu, Wei-Rong Jin, Yu-Dong Xu, Qing-Hua Zhang, Yong-Qing Yang

**Affiliations:** 1 Shanghai Research Institute of Acupuncture and Meridian, Shanghai University of Traditional Chinese Medicine, Shanghai, PR China; 2 National Key Laboratory of Plant Molecular Genetics, Institute of Plant Physiology & Ecology, Chinese Academy of Sciences, Shanghai, PR China; 3 National Engineering Center for Biochips at Shanghai, Shanghai, PR China

## Abstract

**Background:**

Asthma has become an important public health issue and approximately 300 million people have suffered from the disease worldwide. Nowadays, the use of acupuncture in asthma is increasing. This study intended to systematically analyze and compare the gene expression profiles between the asthmatic and acupuncture-treated asthmatic rat lung, and tried to gain insight into the molecular mechanism underlying the early airway response (EAR) phase of asthma treated by acupuncture.

**Methods:**

Four tag libraries of serial analysis of gene expression (SAGE) were established from lung tissues of control rats (CK), asthmatic rats (AS), asthmatic rats treated by acupuncture (ASAC), and control rats treated by acupuncture (CKAC). Bioinformatic analyses were carried out by using the methods including unsupervised hierarchical clustering, functional annotation tool of the database for annotation, visualization, and integrated discovery (DAVID), gene ontology (GO) tree machine, and Kyoto encyclopedia of genes and genomes (KEGG) pathway analysis.

**Results:**

There were totally 186 differentially expressed tags (P < 0.05, P_CK/AS_) between the libraries of CK and AS, 130 differentially expressed tags between libraries of AS/ASAC (P < 0.05, P_AS/ASAC_), and 144 differentially expressed tags between libraries of CK/CKAC (P < 0.05, P_CK/CKAC_). The gene expression profiles of AS and ASAC were more similar than other libraries via unsupervised SAGE clustering. By comparison of P_CK/AS _and P_AS/ASAC_, the DAVID genes functional classification was found to be changed from "immune response" to "response to steroid hormone stimulus", and the GO term "antigen processing and presentation of peptide antigen" disappeared in P_AS/ASAC_. Totally 3 same KEGG pathways were found among the three groups. Moreover, 21 specific tags of the acupuncture in treating asthma were detected using Venn diagrams.

**Conclusion:**

Our SAGE research indicates that the gene expression profile of the EAR phase of asthma could be effectively and specifically regulated by acupuncture, which suggests that the gene expression of immune response and steroid hormone may play an important role in the treatment.

## Background

Asthma is a complex syndrome involving potentially permanent airway obstruction, airway hyperresponsiveness, and multicellular inflammation. It is estimated that approximately 300 million people have suffered from asthma worldwide and the burden of this disease in countries as well as families is increasing [1]. Although inhaled steroids can significantly improve the symptoms, curative therapies are not yet available [2]. Moreover, there are also significant concerns regarding the potential side effects from the long term use of conventional drugs, such as corticosteroids. Thus, an effective, low-risk, and non-drug strategy would provide a valuable and adjunctive treatment in asthma management [3]. Complementary and alternative medicine (CAM), such as traditional Chinese herbal remedies, homeopathy as well as acupuncture, is widely applied in the asthma management. It is reported that CAM has been used among 59% of patients with asthma or rhinosinusitis in the United Kingdom, 41% in the United States, 26.5% in Germany, and 27.2% in Singapore [4].

Acupuncture literally means to puncture with a needle, which is an important therapy in traditional Chinese medicine (TCM) for at least 2,500 years [5]. The theory of acupuncture holds that there are different types of energy flow (qi) in the human body, and the disruptions of qi are believed to be responsible for diseases. Acupuncture practitioners may use thin, solid, metallic needles to correct the imbalances of the flow via the stimulation of special points in the body, which are manipulated manually or by electrical stimulation. The therapy is developed as a relatively global system of medicine and is utilized to treat many diseases. The World Health Organization listed asthma and other 42 indications for acupuncture in 1979 [6] and classified the diseases treated by acupuncture into four categories, 107 illnesses in 2002 [7]. The National Institutes of Health (NIH) has accepted the validity of acupuncture treatment [8] and recommended it as an adjunctive treatment in comprehensive management programs of addiction, stroke rehabilitation, and asthma, etc [5,9]. Acupuncture has traditionally been used to treat asthma in China and has been shown to be beneficial in acute asthma in short term [10,11]. However, well-designed scientific researches in this field are needed and encouraged, which are not only important for elucidating the mechanism, but also useful for exploring new pathways in a systematic manner.

Acupuncture is a complex intervention on diseases and many studies have demonstrated that acupuncture can cause multiple biological responses and regulate many cellular and physiological processes, which could lead to changes of gene expression [5]. These processes could occur at either the proximal or distal ends of acupuncture application, which may be mediated mainly by neural and humoral mechanisms [5,12]. High-throughput technologies, such as microarray and serial analysis of gene expression (SAGE), may help to reveal and clarify the possible mechanism of acupuncture. In the nerve system, acupuncture was reported to regulate brain aging related genes [13], suppress several genes in the nociceptive pathways [14], and up-regulate genes in the spinal cord injury [15] by using microarray. In the immune and endocrine system, results of microarray have revealed that acupuncture could regulate genes to increase activities of natural killer cell [16] and superoxide dismutase [17], to keep the cytokines balance between Th1 and Th2 [18], and to induce hypocholesterolemic effects [19]. However, these researches focused mainly on the nerve and immune systems, and the analyses of the generated data were limited. SAGE, as a powerful expression profiling method and much more accessible for dissecting the complex system, was applied to qualitatively and quantitatively evaluate the transcription of the genes via particular length without the prerequisite of a hybridization probe for each transcript [20]. However, there has been no SAGE report concerning the mechanism of acupuncture in treating asthma so far.

In this study, four SAGE tag libraries were established respectively from lung tissues of control rats, asthmatic rats, asthmatic rats treated by acupuncture, and control rats treated by acupuncture. The study aimed to systematically analyze and compare the gene expression profiles in the lung of the four different groups, and attempted to examine the molecular mechanism of early airway response (EAR) phase of asthma treated by acupuncture.

## Methods

### Animal and experimental asthma model

Pathogen-free, male Sprague-Dawley (SD) rats (4 weeks old, 110–130 g, SLAC Laboratory Animal Co. Ltd., Shanghai, China), raised in a pathogen-free rodent facility and provided with food and water *ad libitum*, were randomly divided into four groups (each group contained 8 rats): control rats (CK), asthmatic rats (AS), asthmatic rats treated by acupuncture (ASAC), control rats treated by acupuncture (CKAC). The protocol of SD rat model of asthma was described as previously [21] and rats of CK and CKAC were sensitized and boosted to normal saline instead of OVA. Rats were kept in animal facilities approved by the Shanghai Committee for Accreditation of Laboratory Animal and the animal experiment conformed to the regulations of the State Science and Technology Commission.

### Acupuncture treatment

**Figure 1:**
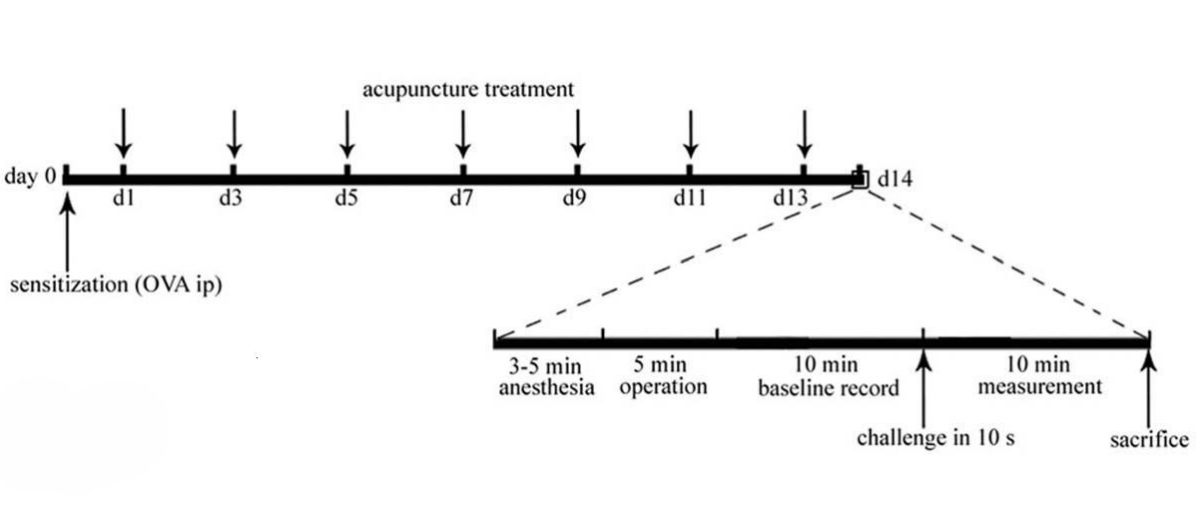
**Protocol and measurements for the rat model of allergic asthma**. Male Sprague-Dawley rats were sensitized intraperitoneally at day 0 with 1 mg of ovalbumin (OVA) precipitated with 10 mg of aluminium hydroxide gel in 1 ml normal saline. Manual acupuncture was performed regularly from the first day after the sensitization and given every other day over a period of two weeks. On day 14, the sensitized animals were challenged with 1 ml/kg of 5% OVA in normal saline (5 mg/kg) by injection into the external jugular vein over 10 s. The respiratory parameters of pulmonary resistance, dynamic compliance and respiratory rate were immediately recorded for 10 min. Rats were killed immediately after the measurement. Rats of CK and CKAC were sensitized and challenged with normal saline instead of OVA. Rats of CKAC received the same acupuncture treatment as those of ASAC.

Manual acupuncture was performed for two weeks from the first day after the sensitization, once every other day (Figure 1). The acupuncture points, Dazhui (GV 14, between C7 and T1 vertebrae), bilateral Fengmen (BL12, foveola laterally between T2 and T3 vertebrae) and bilateral Feishu (BL13, foveola laterally between T3 and T4 vertebrae), were selected based on the theory of TCM in treating asthma [22]. In consideration of the specific and non-specific effects of acupuncture [23], the same points in normal rats were selected and acted as control of ASAC, which could distinguish the specificity of acupuncture in treating asthma. The stainless needles (0.30 × 13 mm, Suzhou Medical Appliance Factory, Suzhou, China) were inserted about 5 mm deep into the skin. The needles were twisted approximately 360° evenly at the rate of 60 times/min for 20 s, manipulated every 5 min and withdrawn after 20 min. For the convenient manipulation of the acupuncture points on the back, rat was placed on the suspended shelf (50 × 45 mm, about 50 cm high from the ground, see additional file 1), which could easily make it calm and stand still without anesthesia. Acupuncture was performed by the same experienced practitioner and the animals were handled while awake, with special care to minimize stress. Both rats in ASAC and CKAC received the same acupuncture treatment.

### Measurement of pulmonary function

The measurements of pulmonary resistance (RL), dynamic compliance (Cdyn), and respiratory rate (RR), were modified from Glaab T *et al *[24,25]. Briefly, a rat was placed in supine position on a wood plate warmed by an incandescent lamp after anaesthesia. At the upper part of the trachea, a T-shape cutting was made and a T-shape cannula, which was directly attached to a heater controlled pneumotachograph (Series 3850A, Hans Rudolph, USA), was gently inserted into the trachea. Tidal flow was determined by the pneumotachograph connected to a differential pressure transducer (600D-011, AutoTran, USA). To measure transpulmonary pressure, a water-filled PE-90 tubing was inserted into the esophagus to the level of the midthorax (lower one-third of the esophagus) and coupled to a pressure transducer (PT14MX, Jialong Teaching Equipment, Shanghai). The pneumotachograph tidal flow signal was integrated with time to obtain tidal volume. RL and Cdyn were calculated over a complete respiratory cycle using an integration method over flows, volumes and pressures, and were continuously recorded with software (Shanghai Medical College, Fudan University) for physiology experiments. Respiratory parameters were averaged in 60 s segments and maximum RL, minimum Cdyn and change of RR values were taken and calculated as differential values subtracted from the corresponding baseline values (Figure 1). Then the rats were sacrificed. The lungs were excised right away, rinsed in ice-cold normal saline, dissected free from surrounding tissues in an ice-bath and frozen immediately in liquid nitrogen.

### Construction, annotation, and confirmation of the SAGE libraries

Construction and annotation of the SAGE libraries were described as previously [21]. The confirmation of the four SAGE libraries was performed by Quantitative Real-Time PCR (qRT-PCR) on an Applied Biosystems 7300 Real-Time PCR System using TOYOBO Realtime PCR Master Mix (Toyobo, Osaka, Japan). The threshold cycle number was determined using SDS v1.4 Software and the reactions were performed in triplicate. Total RNA (5 μg) was reversely transcribed into cDNA by using the RevertAid First Strand cDNA synthesis kit (Cat. No. K1622; Fermentas, EU). For qRT-PCR of the cDNA, primer pairs were designed to generate intron-spanning products of 101–150 bp (Primer sequences were listed in additional file 2). The generation of specific PCR products was confirmed by the melting curve and gel analysis. The expressional ratio was calculated according to the formula 2^(Rt-Et)^/2^(Rn-En) ^as described previously [26]. Transcripts with a twofold increase in expression were considered to be up-regulated and those with a 0.5-fold decrease in expression were considered to be down-regulated.

### Bioinformatic analysis of SAGE tags

To identify genes preferentially regulated in the four groups, the two-way unsupervised clustering method based on tag copies was applied. This unguided approach allowed pattern discovery for subsequent supervised functional analysis. To reduce the magnitude effects of the extreme data, the genes with total tag counts less than 20 were filtered. The dendrogram of differentially expressed tags was created by using the TIGR MultiExperiment Viewer 4.0 [27], mainly with the average clustering and Euclidean distance.

Differentially expressed genes (P < 0.05) between SAGE libraries were functionally annotated and classified by using the functional annotation tool of database for annotation, visualization, and integrated discovery (DAVID) [28], which provided integrated solutions for the annotation and analysis of genome-scale datasets derived from high-throughput technologies.

Key regulatory processes in asthma were analyzed by Gene Ontology (GO) Tree Machine [29]. GO Tree Machine generated a directed acyclic graph (DAG) for input gene sets, which was made to identify the most important GO categories and to suggest their potential biological importance.

The Kyoto encyclopedia of genes and genomes (KEGG) pathway is a collection of manually drawn pathway maps of the molecular interaction and reaction networks. The KEGG pathways of the differentially expressed genes between SAGE libraries were matched by using the DAVID Functional Annotation Tool.

### Statistical analysis

One-way ANOVA (analysis of variance) followed by the least significant difference (LSD) test for post hoc analysis was used to analyze the significance of RL, Cdyn and RR among the four groups. Statistical analysis for the significance of each of the four SAGE libraries was made using Monte Carlo analysis. The enrichments of GO Tree Machine were statistically significant as determined by the hypergeometric test [29].

## Results

### Measurements of pulmonary functions after OVA challenge

The allergen-specific early airway response to ovalbumin (OVA) in sensitized rats showed significant increases in RL and significant decreases in simultaneously measuring Cdyn and RR compared with those of the controls, thus indicating an allergen-specific EAR to OVA. By One-way ANOVA among the four groups, the significant differences (P < 0.05) were at 1–7 min for RL, 2 and 5 min for Cdyn, 2–4 min for RR after challenge. The LSD test for post hoc analysis indicated that RL in ASAC group was significantly decreased at 1–4 min after challenge in comparison with AS group (P < 0.05, Table 1), and Cdyn and RR in ASAC group were significantly increased at 2, 5 min and 2, 3 min respectively after challenge when compared with those of AS group (P < 0.05, see additional file 3). The results indicated that the immediate effects (1–3 min) of acupuncture were the most significant in the EAR phase of asthma.

**Table 1:** The comparisons of pulmonary resistance (kPa/ml/s) of the four groups

Groups	Immediate effects			Early effects				Recovery effects		
			
	Min 1	Min 2	Min 3	Min 4	Min 5	Min 6	Min 7	Min 8	Min 9	Min 10
CK	0.0003 ± 0.0038	0.0010 ± 0.0059	0.0085 ± 0.0237	0.0064 ± 0.0119	0.0065 ± 0.0160	0.0039 ± 0.0127	0.0062 ± 0.0120	0.0037 ± 0.0074	0.0055 ± 0.0090	0.0066 ± 0.0105
AS	0.0317 ± 0.0394*	0.1969 ± 0.1051*	0.2363 ± 0.1197*	0.1874 ± 0.0897*	0.1292 ± 0.0787*	0.0893 ± 0.0575*	0.0622 ± 0.0512*	0.0416 ± 0.0393	0.0194 ± 0.0272	0.0062 ± 0.0205
ASAC	-0.0016 ± 0.0153#	0.0283 ± 0.0539#	0.0695 ± 0.0716#	0.0762 ± 0.0611#	0.0810 ± 0.0764	0.0567 ± 0.0543	0.0359 ± 0.0564	0.0187 ± 0.0425	0.0124 ± 0.0373	0.0025 ± 0.0258
CKAC	-0.0025 ± 0.0049	-0.0025 ± 0.0071	0.0011 ± 0.0101	0.0013 ± 0.0081	0.0007 ± 0.0098	0.0008 ± 0.0077	0.0018 ± 0.0082	0.0036 ± 0.0088	0.0054 ± 0.0076	0.0027 ± 0.0103

Data were shown as mean ± SD (n = 8). The values of pulmonary resistance in the table were expressed as differential values subtracted from the corresponding baseline values. Statistical comparisons were made by ANOVA with LSD test for post hoc analysis. * indicate P < 0.05, compared to CK; # indicate P < 0.05, compared to AS.

CK: control rats; AS: asthmatic rats; ASAC: asthmatic rats treated by acupuncture; CKAC: control rats treated by acupuncture.

### General analysis of SAGE libraries

The four SAGE libraries of rat lung were deposited in the SAGEmap database at National Center for Biotechnology Information (, Accession numbers are GSM45195, GSM119459, GSM279944, and GSM279945). The information of the matched genes and ESTs of the 4 libraries was listed in the Table 2.

**Table 2:** Summary of serial analysis of gene expression data for the four libraries

SAGE tag	CK	AS	ASAC	CKAC
Total tags	28,284	26,552	26,772	29,284
Unique tags	12,857	12,221	11,656	12412
Genes matched	54.1%	55.5%	51.2%	50.5%
ESTs matched	38.5%	36.2%	16.9%	17.0%
No matched	7.4%	8.3%	31.9%	32.5%

CK: control rats; AS: asthmatic rats; ASAC: asthmatic rats treated by acupuncture; CKAC: control rats treated by acupuncture; EST: expressed sequence tag.

By Comparing with the SAGE data between the libraries of CK and AS, there were totally 186 differentially expressed tags (P < 0.05, P_CK/AS_). Similarly, there were 130 differentially expressed tags between libraries of AS and ASAC (P < 0.05, P_AS/ASAC_), and 144 differentially expressed tags between libraries of CK and CKAC (P < 0.05, P_CK/CKAC_). See additional file 4 for the lists of differentially expressed tags of P_CK/AS_, P_AS/ASAC_, P_CK/CKAC_.

### Confirmation of SAGE Results by qRT-PCR

**Figure 2:**
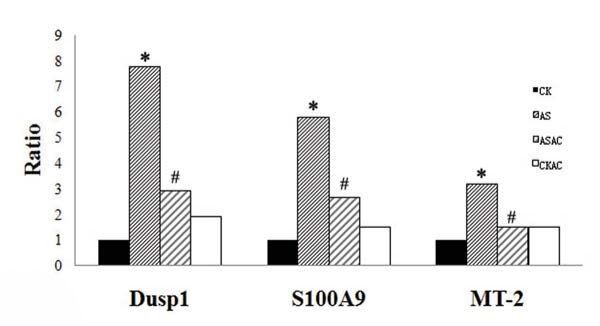
**Real-time PCR confirmation of differentially regulated genes of interest predicted by SAGE**. * meant the expression levels were up-regulated more than 2 folds compared with CK, # meant the expression levels were down-regulated more than 0.5 fold compared with AS. The expression levels of the three genes of CKAS were up-regulated less than 2 folds compared with CK.

To confirm the expression profiles among the four SAGE libraries, three differentially expressed genes of interest were chosen and their expression levels were evaluated by qRT-PCR. Dusp1 encodes a protein that catalyzes the dephosphorylation and inactivation of MAP kinase. S100A9 is a calcium binding protein that may be associated with acute inflammatory processes. MT-2 has a high content of cysteine residues that bind various heavy metals and could be transcriptionally regulated by both heavy metals and glucocorticoids. The results indicate that the expression profiles of representative genes in qRT-PCR analysis correspond to the SAGE data (Figure 2), which supports the validation of our quantitative data for further bioinformatic analysis.

### Unsupervised SAGE clustering based on tag expression

**Figure 3:**
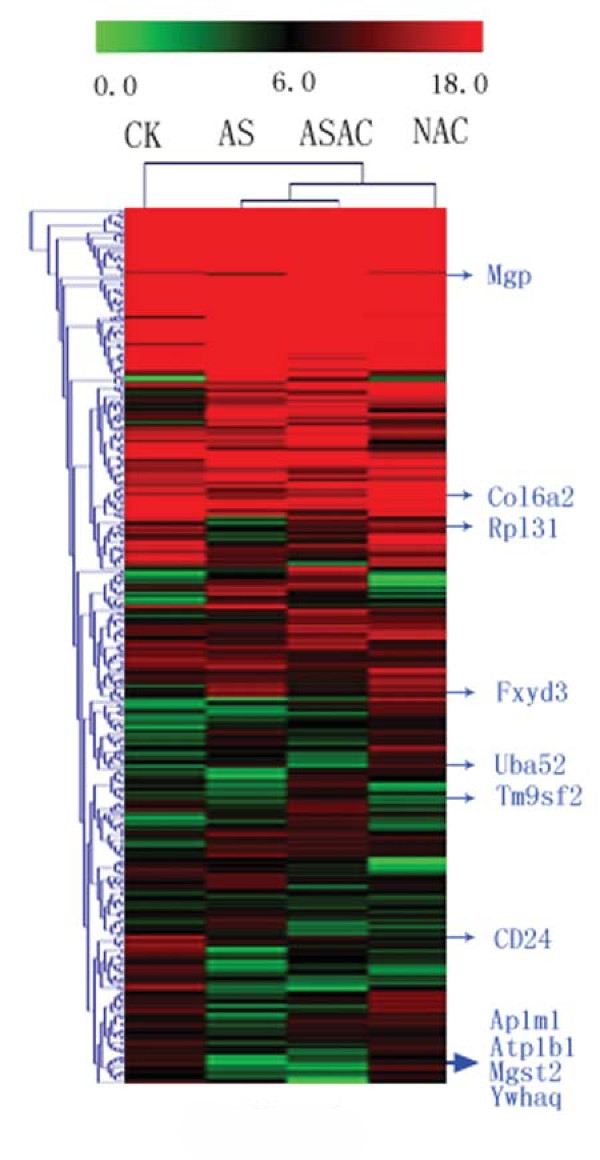
**The dendrogram of the hierarchical cluster analysis**. The genes with over fivefold change or less than 0.2-fold between AS and ASAC were listed at the right side of the graph. Abbreviations of the seven up-regulated genes: Fxyd3: FXYD domain-containing ion transport regulator 3; Col6a2: procollagen, type VI, alpha 2; Uba52: ubiquitin A-52 residue ribosomal protein fusion product 1; Tm9sf2: transmembrane 9 superfamily member 2; CD24: CD24 antigen; Mgp: matrix Gla protein; Rpl31: ribosomal protein L31. Abbreviations of the four down-regulated genes: Ap1m1: Adaptor-related protein complex AP-1, mu subunit; Mgst2: Microsomal glutathione S-transferase 2; Atp1b1: ATPase, Na+/K+ transporting, beta 1 polypeptide; Ywhaq: tyrosine 3-monooxygenase/tryptophan 5-monooxygenase activation protein, theta polypeptide.

Based on the global expression matrix, the dendrogram clearly identified CK from the other 3 libraries, while AS and ASAC displayed more similar patterns than CKAC on the gene expression profiles (Figure 3). The genes with over fivefold change or less than 0.2-fold between AS and ASAC were listed at the right side of the hierarchical clustering dendrogram. Seven genes were ranked with an expression over 5-fold increase: Fxyd3 (Rn.3896), Col6a2 (Rn.11889), Uba52 (Rn.4300), Tm9sf2 (Rn.11839), CD24 (Rn.6007), Mgp (Rn.2379), and ribosomal protein L31 (Rpl31, Rn.1101). Four genes were listed with an expression over 5-fold decrease: Ap1m1 (Rn.139185), Mgst2 (Rn.7854), Atp1b1 (Rn.8925), and Ywhaq (Rn.2502).

### The comparisons of the DAVID gene functional classification of P_CK/AS_, P_AS/ASAC _and P_CK/CKAC_

**Figure 4:**
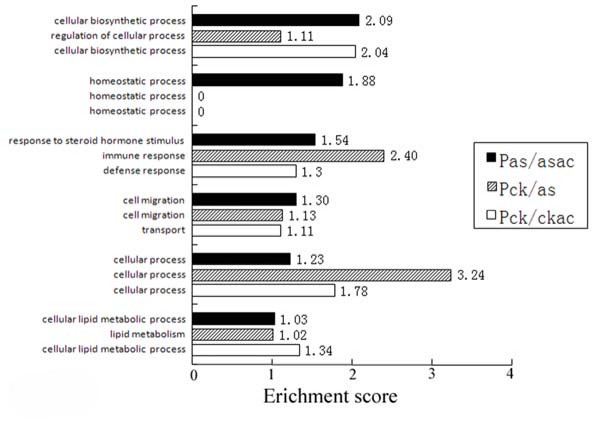
**The comparison of the selected DAVID gene functional classification among P_CK/AS_, P_AS/ASAC _and P_CK/CKAC_**. Six DAVID gene functional classifications with enrichment score equal to or higher than 1 were selected. There is no classification homeostatic process in P_CK/AS _and P_CK/CKAC_, so the enrichment score is zero.

Six DAVID gene functional classifications of P_AS/ASAC _with the enrichment score equal to or higher than 1.0 were found. They were cellular biosynthetic process, homeostatic process, response to steroid hormone stimulus, cell migration, cellular process, and cellular lipid metabolic process. The related gene functional classifications among P_CK/AS_, P_AS/ASAC _and P_CK/CKAC _were compared (Figure 4). The classification of homeostasis process was only found in P_AS/ASAC_. The DAVID gene functional classification was found to be changed from "immune response" in P_CK/AS _to "response to steroid hormone stimulus" in P_AS/ASAC_. (See additional file 5 for the gene lists of DAVID gene functional classification of P_CK/AS_, P_AS/ASAC_, P_CK/CKAC_)

### DAG analysis of gene categories of P_CK/AS_, P_AS/ASAC _and P_CK/CKAC_

**Figure 5:**
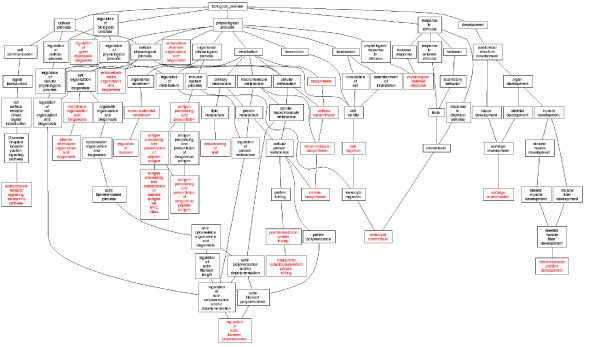
**The DAG graphs of the P_CK/AS_**. After uploading 186 differentially expressed tags between the libraries of CK and AS to the GO Tree Machine, the directed acyclic graph was generated automatically. Totally 25 enriched GO categories of biological process were found in P_CK/AS_.

**Figure 6:**
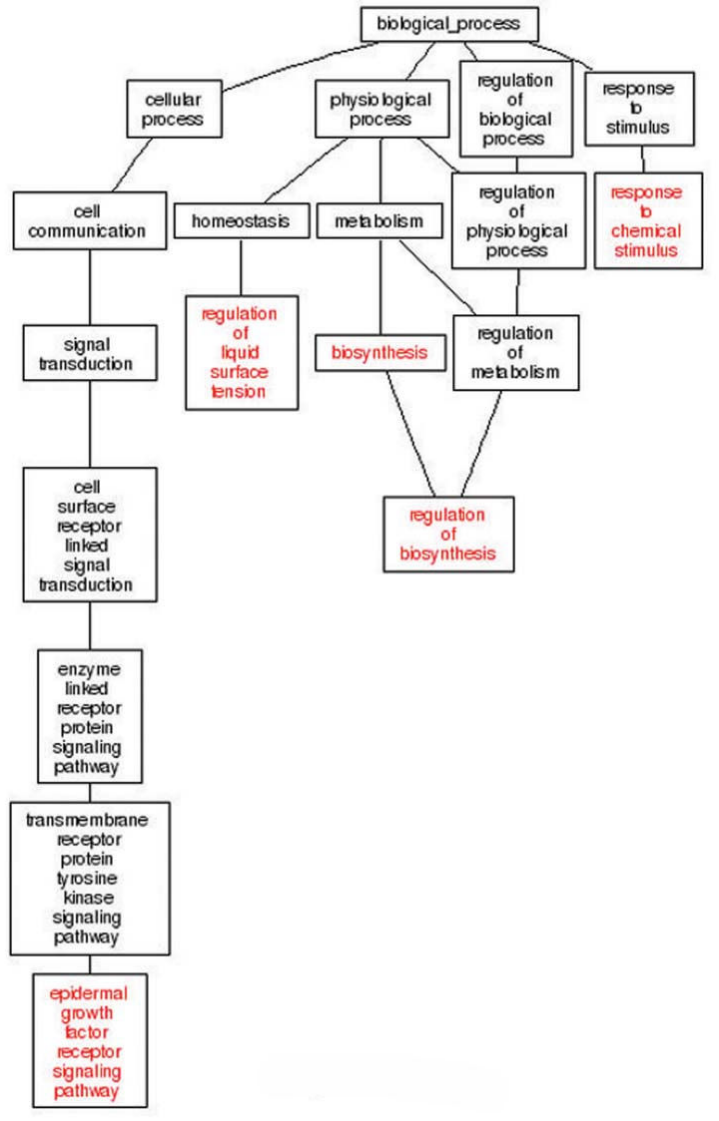
**The DAG graphs of the P_AS/ASAC_**. 130 differentially expressed tags between libraries of AS and ASAC were uploaded to the GO Tree Machine and totally 5 enriched GO categories of biological process were found in P_AS/ASAC_.

**Figure 7:**
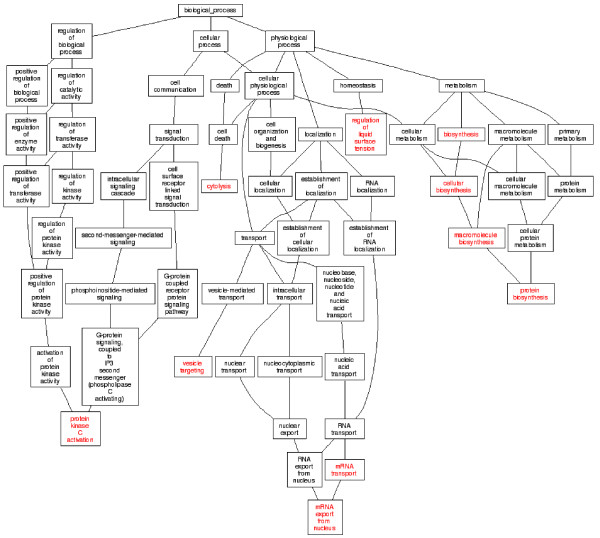
**The DAG graphs of the P_CK/CKAC_**. 144 differentially expressed tags between libraries of CK and CKAC have matched 10 enriched GO categories of biological process by using the GO Tree Machine.

There were 25, 5 and 10 enriched GO categories of biological process in P_CK/AS_, P_AS/ASAC _and P_CK/CKAC _respectively (Figure 5, 6, 7). Biosynthesis was only one overlapping GO term among them. The GO terms, such as antigen processing and presentation, cell migration, acetylcholine receptor signaling, muscarinic pathway disappeared in P_AS/ASAC_, and new GO terms, like regulation of biosynthesis, regulation of liquid surface tension, epidermal growth factor receptor signaling pathway appeared in P_AS/ASAC _when compared with P_CK/AS_. Regulation of liquid surface tension was the only shared GO term between P_AS/ASAC _and P_CK/CKAC_.

### Finding KEGG pathways of P_CK/AS_, P_AS/ASAC _and P_CK/CKAC_

For understanding functional roles of the differentially expressed genes, KEGG pathway analysis was assigned by applying the DAVID annotation tool. There were 15, 8, and 2 matched KEGG pathways (counts ≥ 3) in P_CK/AS_, P_AS/ASAC _and P_CK/CKAC _respectively (see Table 3), in which tight junction pathway kept the same. Three pathways, MAPK signaling pathway, T cell receptor signaling pathway, focal adhesion, were identical between P_CK/AS _and P_AS/ASAC_. Although there are the same matching pathways among three groups, the involved genes were different. (See additional file 6)

**Table 3:** Kyoto encyclopedia of genes and genomes (KEGG) pathways of differentially expressed tags

KEGG pathways	P_CK/AS_	P_AS/ASAC_	P_CK/CKAC_
Tight junction	X	X	X
MAPK signaling pathway	X	X	
T cell receptor signaling pathway	X	X	
Focal adhesion	X	X	
B cell receptor signaling pathway	X		
Toll like receptor signaling pathway	X		
ECM receptor interaction	X		
WNT signaling pathway	X		
Cell communication	X		
Leukocyte transendothelial migration	X		
Regulation of actin cytoskeleton	X		
Adherens junction	X		
FCεRI signaling pathway	X		
Antigen processing and presentation	X		
Axon guidance	X		
Insulin signaling pathway		X	
GnRH signaling pathway		X	
ErbB signaling pathway		X	
Gap junction		X	
SNARE interactions in vesicular transport			X

P_CK/AS_: 186 differentially expressed tags between the libraries of control rats and asthmatic rats; P_AS/ASAC_: 130 differentially expressed tags between libraries of asthmatic rats and asthmatic rats treated by acupuncture; P_CK/CKAC_: 144 differentially expressed tags between libraries of control rats and control rats treated by acupuncture; MAPK: Mitogen-activated protein kinase; ECM: Extracellular matrix; WNT: Wingless; FCεRI: Fc epsilon receptor I; GnRH: Gonadotropin-releasing hormone; SNARE: Soluble n-ethylmaleimide-sensitive-factor attachment protein receptor.

### The Venn diagram among P_CK/AS_, P_AS/ASAC_, and P_CK/CKAC_

**Figure 8:**
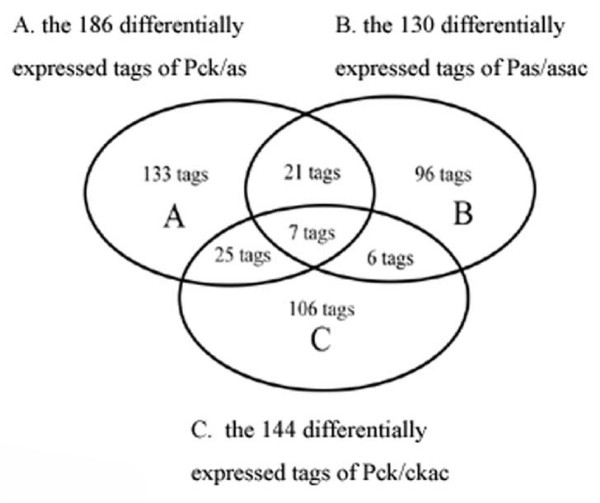
**The Venn diagram among P_CK/AS_, P_AS/ASAC_, and P_CK/CKAC_**. The diagram shows 21 common tags between P_CK/AS _and P_AS/ASAC_, 7 common tags in the three groups, 25 common tags between P_CK/AS _and P_CK/CKAC_, and 6 common tags between P_AS/ASAC _and P_CK/CKAC_.

By using the Venn diagram, 21 tags were attributed to the same group between P_CK/AS _and P_AS/ASAC_, 7 tags were found involved in the three groups, 6 tags were identified between P_AS/ASAC _and P_CK/CKAC_, and 25 tags were commonly clustered between P_CK/AS _and P_CK/CKAC_(Figure 8). (See additional file 7 for the gene lists of each group)

## Discussion

The gene expression profiles of acupuncture in treating asthma were not yet well studied before. Our SAGE study, in which acupuncture served as a kind of biological perturbation of systems biology, has provided a molecular base of EAR phase of asthma treated by acupuncture and may provide clues for the further research.

### The cluster analysis of gene expression profiles of the four libraries and genes with marked changes

Based on the global expression matrix, the result of the hierarchical cluster analysis indicated that the gene expression profiles of different libraries changed a lot when receiving different perturbation, such as OVA sensitization and acupuncture. It suggested that acupuncture could perturb the biological condition by regulating a number of genes. Moreover, the gene expression profiles of ASAC and CKAC were different, and the profiles of AS and ASAC were more similar than those in other groups, which indicated the specific effect of acupuncture in the EAR phase of asthma.

The genes with marked changes in expression levels probably have potential importance in treating asthma. CD24, referred to as heat-stable antigen, is an important co-stimulatory molecule in immunity [30]. Previous studies have suggested that CD24 was involved in cell adhesion and signal transduction via phosphorylation of intracellular proteins, intracellular calcium mobilization, and activation of transcription factors [31]. In our libraries, the expression of CD24 was found to be down-regulated 12 times in asthma while up-regulated 7 times by acupuncture. The result suggested that the gene may correlate with the immune-modulating effects of acupuncture in EAR phase of asthma.

### The gene expressions of immune response and steroid hormone were regulated by acupuncture in the EAR phase of asthma

Several immune-related GO terms, such as antigen processing and presentation, antigen processing and presentation of peptide antigen, antigen processing and presentation of exogenous peptide antigen, disappeared in P_AS/ASAC _via GO Tree Machine. Moreover, the DAVID genes functional classification was found to shift from "immune response" in P_CK/AS _to "response to steroid hormone stimulus" in P_AS/ASAC_. The changes suggested that the immune response of EAR phase of asthma was attenuated by acupuncture, which may be realized through the release of the endogenous steroid hormone.

There were 14 genes in the DAVID genes functional classification "response to steroid hormone stimulus" in P_AS/ASAC_, in which the genes of MGP (Rn.2379) and Srd5a2 (Rn.9938) were interesting. MGP is a vitamin K-dependent protein that serves as a substrate for the enzyme γ-carboxylase [32]. It plays a role in lung growth and development [33] and its expression was incited by dexamethasone [34]. In our libraries, the expression of MGP was up-regulated by acupuncture, which may result from the release of endogenous steroid hormones in consideration of the DAVID gene classification. Srd5a2, an isozyme of the 5-alpha-reductase family, is present in a large number of cells. It plays a key role in the conversion of testosterone to dihydrotestosterone and in the removal of excess of potentially neurotoxic steroids [35]. In our libraries, the expression of Srd5a2 was up-regulated in asthma but down-regulated by acupuncture. The changed expression suggested that Srd5a2 served as an adjustable gene target of acupuncture, which may relate with the genesis of steroid by acupuncture in the EAR phase of asthma.

### Pathways regulated by acupuncture in the EAR phase of asthma

Pathway-level visualization of omics data provides an essential means for systems biology to capture the systematic properties of the inner activities of cells. Eight pathways were involved in the P_AS/ASAC_, in which tight junction pathway was commonly shared among P_CK/AS_, P_AS/ASAC_, and P_CK/CKAC_. Three pathways were the same between P_CK/AS _and P_AS/ASAC_, and 4 pathways were unique in P_AS/ASAC_.

The tight junction pathway was considered to be the background of OVA sensitization and acupuncture regulation. MAPK cascade is one of the three same pathways between P_CK/AS _and P_AS/ASAC_. It contributes to the amplification and specificity of the transmitted signals and plays discrete yet complementary roles in accentuating allergic airway inflammation [36]. It is also reported that the MAPK pathway could regulate steroidogenesis [37]. GnRH signaling pathway is one of the four unique pathways in P_AS/ASAC_, which is the central regulator of the reproductive hormonal cascade [38] and could enhance the basal steroidogenesis [39]. Several signaling pathways are activated by GnRH, including MAPK, and protein kinase C [40], which could lead to the increase of corticotropin-releasing hormone-binding protein at mRNA level [41]. In our study, the data suggested that GnRH signaling pathway may interact with other pathways and participate in the genesis of steroid by acupuncture in the EAR phase of asthma.

### The specific and non-specific tags of acupuncture

From the Venn diagrams, the 21 tags were found to represent the specific genes of acupuncture in treating asthma. By using the DAVID classification analysis tool, 18 of the 21 tags were divided into 7 groups. The classification with highest score was "response to steroid hormone stimulus" (enrichment score = 1.71), which was in accordance with the classification of the P_AS/ASAC_. The genes involved in the classification were: MGP, Abca2, and Sult1a1, which demonstrated that the transportation and production of steroid hormone may be regulated by acupuncture in the EAR phase of asthma at the level of transcription.

Abca2 expressed in a broad range of tissues and previous studies indicated that the gene participated in the transport of steroids [42]. Besides, the elevated expression of Abca2 was considered to be a conserved mechanism of cell survival [43]. In our study, Abca2 was up-regulated in asthma, which may correlate with the cell survival in the EAR phase of asthma. Sult1a1 is a member of sulfotransferase families, primarily localized in the trans-Golgi apparatus and associated with the sulfation of steroids and various hormones [44,45]. Our SAGE data demonstrated that Sult1a1 was up-regulated in asthma, which indicated the gene may catalyze the sulfation process of steroidgensis. However, the two genes both belong to the DAVID classification "homeostatic process" and the gene expressions of Abca2 and Sult1a1 after acupuncture were almost equivalent to those of the controls. It suggested that acupuncture could alleviate the OVA challenge by maintaining the internal equilibrium.

Seven common tags were found among the different groups when receiving the chemical and acupuncture stimulation both in normal and asthmatic conditions. Twenty-five tags were found to be the same when receiving the OVA sensitization or acupuncture treatment. Furthermore, 6 tags were identified as the non-specific genes of acupuncture in P_AS/ASAC _and P_CK/CKAC_. The above-mentioned genes belong mainly to cellular process, and cell communication, which suggested that they may serve as the background genes in EAR phase of asthma and acupuncture. For example, Syntaxin 5 is a Golgi-localized SNARE protein required for endoplasmic reticulum-Golgi traffic in yeast and Golgi reassembly following cell division in mammalian cells [46].

## Conclusion

We have presented evidences of gene expression profiles in the lung of asthmatic rats and those treated by acupuncture via SAGE. The study indicates that the gene expression profile of the EAR phase of asthma could be effectively and specifically regulated by acupuncture at the transcriptional level, which suggests that the gene expression of immune response and steroid hormone may play an important role in the treatment.

## Competing interests

The authors declare that they have no competing interests.

## Authors' contributions

YL was responsible for the qRT-PCR validation, bioinformatic analyses and draft of the manuscript. JG, WY, LY, & JW duplicated the rat asthmatic model and established four SAGE tag libraries, WY, ZQ, XY, & YY contributed to the design of the study and draft of the manuscript. All authors read and approved of the final manuscript.

## Supplementary Material

Additional file 1 **Rat on the suspended shelf**. For the convenient manipulation of the acupuncture points on the back, rat was placed on the suspended shelf, which could easily make it calm and stand still without anesthesia. Click here for file

Additional file 2 **The intron-spanning primer pairs of genes of quantitative real-time PCR confirmation**. The primer pairs of 3 differentially expressed genes of interest (Dusp1, S100A9, and MT-2) and the reference gene (GAPDH). Click here for file

Additional file 3 **The comparisons of Cdyn and RR of the four groups**. The table included the 10 min measurement of Cdyn and RR of the four groups. Click here for file

Additional file 4 **The lists of differentially expressed tags of P_CK/AS_, P_AS/ASAC_, P_CK/CKAC_**. The details of the 186, 130 and 144 differentially expressed tags (P < 0.05) of the P_CK/AS_, P_AS/ASAC_, and P_CK/CKAC_. This table included the tag sequence, tag copies of different libraries, UniGene number and the gene description. Click here for file

Additional file 5 **The gene lists of DAVID gene functional classification of P_CK/AS_, P_AS/ASAC_, P_CK/CKAC_**. The complete gene lists of 6 functional groups with enrichment scores equal to or higher than 1 were shown for the Figure 4. Click here for file

Additional file 6 **The gene lists of the KEGG pathways**. The complete gene lists of the matched KEGG pathways were demonstrated for the Table 3. Click here for file

Additional file 7 **The gene lists of Venn diagram**. The details of the different gene groups of Venn diagram, which included the tag sequence, tag copies of different libraries, UniGene number and the gene description. Click here for file
