# Supplementary material for: Use of serial analysis of gene expression to reveal the specific regulation of gene expression profile in asthmatic rats treated by acupuncture
Source: J Biomed Sci. 2009 May 6;16(1):46. doi: 10.1186/1423-0127-16-46 (PMC2698896; doi:10.1186/1423-0127-16-46)
Supplement: Additional file 2 — The intron-spanning primer pairs of genes of quantitative real-time PCR confirmation. The primer pairs of 3 differentially expressed genes of interest (Dusp1, S100A9, and MT-2) and the reference gene (GAPDH). [file 1423-0127-16-46-S2.doc]

Primer sequences of the Real-Time PCR

| Genes | Sequences (5'to3') |
| --- | --- |
| Dusp1 forword | TTCAAAGCCCCATCACAACC |
| Dusp1 reverse | TTGCATTGCTCCTCCCATG |
| S100A9 forword | ACCCTGAACAAGGCGGAATT |
| S100A9 reverse | TTTGTGTCCAGGTCCTCCATG |
| MT-2 forword | TGCAAGAAAAGCTGCTGTTCC |
| MT-2 reverse | TTACACCATTGTGAGGACGCC |
| GAPDH forword | TCCTGCACCACCAACTGCTTAG |
| GAPDH reverse | AGTGGCAGTGATGGCATGGACT |
