# Supplementary material for: Use of serial analysis of gene expression to reveal the specific regulation of gene expression profile in asthmatic rats treated by acupuncture
Source: J Biomed Sci. 2009 May 6;16(1):46. doi: 10.1186/1423-0127-16-46 (PMC2698896; doi:10.1186/1423-0127-16-46)
Supplement: Additional file 3 — The comparisons of Cdyn and RR of the four groups. The table included the 10 min measurement of Cdyn and RR of the four groups. [file 1423-0127-16-46-S3.doc]

**Additional file 3 The comparisons of Cdyn and RR of the four groups**

| **Respiratory**  **parameters** | **Groups** | **Immediate effects** | | |  | **Early effects** | | | |  | **Recovery effects** | | |
| --- | --- | --- | --- | --- | --- | --- | --- | --- | --- | --- | --- | --- | --- |
|  |  | **Min 1** | **Min 2** | **Min 3** | **Min 4** | | **Min 5** | **Min 6** | **Min 7** | **Min 8** | | **Min 9** | **Min 10** |
| **Cdyn**  **(ml/kPa)** | **CK** | **1.6500±3.7690** | **1.3035±2.4946** | **2.8411±4.4637** | **3.2300±1.7133** | | **3.9699±2.3130** | **3.2303±1.8912** | **4.5434±1.4766** | **3.2564±3.2265** | | **4.3249±3.1654** | **3.5890±2.4511** |
|  | **AS** | **2.7700±10.2000** | **-0.9374±6.7292** | **-1.4176±6.3018** | **-1.3067±8.7967** | | **-2.9211±3.5576*** | **-0.9197±8.1917** | **-1.6942±8.9999** | **-1.7601±9.5179** | | **0.8453±12.3396** | **1.8816±14.0549** |
|  | **ASAC** | **4.3400±7.8320** | **7.2765±9.2110#** | **4.9451±9.8859** | **3.9960±8.9436** | | **3.6144±7.4791#** | **2.5973±5.1534** | **3.5018±11.3961** | **4.0362±14.1387** | | **3.7161±10.7019** | **0.5922±8.0618** |
|  | **CKAC** | **2.6100±4.3170** | **-1.9093±4.6247** | **-0.8128±5.2355** | **-1.4645±4.1846** | | **-2.0271±3.7924** | **-2.3911±4.4499** | **-0.9768±5.5505** | **-2.6381±3.7858** | | **-2.6358±4.6001** | **-4.2110±3.4059** |
| **RR**  **(breaths/min)** | **CK** | **-3.0834±7.2413** | **-3.5749±8.6230** | **-3.9756±7.7349** | **-3.1443±7.6713** | | **-4.0005±6.3185** | **-4.7654±6.2550** | **-2.7611±5.6802** | **-3.0767±5.4893** | | **-2.1713±5.0210** | **-4.2722±5.7779** |
|  | **AS** | **-11.3087±20.7019** | **-26.6341±25.2942*** | **-23.2221±11.3187*** | **-18.4691±11.5075*** | | **-15.2196±11.0295** | **-12.5738±9.5172** | **-10.4202±9.6511** | **-9.2431±12.3202** | | **-7.9799±12.3459** | **-6.3643±12.6144** |
|  | **ASAC** | **-2.7422±16.9278** | **-10.0489±17.2774#** | **-5.8894±17.6432#** | **-12.5987±14.9533** | | **-8.4721±18.5408** | **-13.7058±16.2754** | **-15.5218±17.1236** | **-12.5973±17.3240** | | **-10.3386±17.4715** | **-8.3178±16.0734** |
|  | **CKAC** | **-2.7422±16.9278** | **-10.0489±17.2774** | **-5.8894±17.6432** | **-12.5987±14.9533** | | **-8.4721±18.5408** | **-13.7058±16.2754** | **-15.5218±17.1236** | **-12.5973±17.3240** | | **-10.3386±17.4715** | **-8.3178±16.0734** |

**Data were shown as mean ± SD (n=8). The values of pulmonary resistance in the table were expressed as differential values subtracted from the corresponding baseline values. Statistical comparisons were made by ANOVA with LSD test for post hoc analysis. * indicate P<0.05, compared to CK; # indicate P<0.05, compared to AS. CK: control rats; AS: asthmatic rats; ASAC: asthmatic rats treated by acupuncture; CKAC: control rats treated by acupuncture; Cdyn: dynamic compliance; RR: respiratory rate.**
